# The global impact of COVID‐19 on acute stroke care

**DOI:** 10.1111/cns.13442

**Published:** 2020-07-29

**Authors:** Renyu Liu, Jing Zhao, Marc Fisher

**Affiliations:** ^1^ Department of Anesthesiology and Critical Care Perelman School of Medicine University of Pennsylvania Philadelphia PA USA; ^2^ Department of Neurology Minhang Hospital Fudan University Shanghai China; ^3^ Department of Neurology Beth Israel Deaconess Medical Center Harvard Medical School Boston MA USA

**Keywords:** awareness, COVID‐19, global impact, stroke, stroke care

Prehospital delay has been a major hurdle across the world that impedes optimal stroke care, leading to low thrombolysis and thrombectomy rates in many places. During the present pandemic of the novel coronavirus disease (COVID‐19), with the call for social distancing, patients are less willing to come to the hospital despite the potential devastating consequences of stroke. A recent article from Kansagra AP et al demonstrated that the number of patients who received evaluation for stroke dropped approximately 39% in the United States, indicating a significant decrease in patients seeking acute stroke care.^1^ We observed a similar drop (40%) in stroke admissions across China during the peak period of the COVID‐19 epidemic in 2020 in China, as compared to that observed in the pre‐COVID‐19 period.^2^ The absolute number of thrombolysis and thrombectomy cases dropped 27% and 25%, respectively. Through a survey conducted by the World Stroke Organization (WSO), we found the global average decrease in stroke admissions was approximately 42% as indicated in Table 1 (only these with more than two responses in a given country were reported in this table). While only 7 stroke centers responded to the survey from the United States, the average decrease in stroke admissions (39%) matched the drop reported in the report from Kansagra AP et al^1^ A recent study from Spain reported a similar drop (25%‐30% drop in stroke admission) as in the WSOs survey.^3^ While different countries use different strategies to combat the COVID‐19 pandemic in totally different medical systems and cultural environments, a similar drop of stroke admission was observed, reflecting the similarity of the impact of the COVID‐19 pandemic on stroke care and may reflect an overall lack of stroke awareness and a reluctance to seek medical care.

**TABLE 1:** Stroke admission drops in various countries

	Number of Answers	Mean (%)	Minimum (%)	Maximum (%)	Range (%)
Australia	4	42 ± 41	0	80	80
Austria	2	9 ± 13	0	18	18
Belgium	4	40 ± 12	25	50	25
Brazil	4	60 ± 20	36	85	49
Canada	7	37 ± 28	0	75	75
Colombia	3	33 ± 5	30	38	7.9
France	2	23 ± 11	15	30	15
Germany	5	35 ± 31	0	80	80
Greece	4	50 ± 24	30	80	50
India	4	61 ± 19	40	80	40
Italy	5	42 ± 15	20	60	40
Japan	2	64 ± 37	37	90	53
Philippines	3	73 ± 20	50	85	35
Spain	4	31 ± 24	0	50	50
UK	8	48 ± 17	20	80	60
Ukraine	3	23 ± 12	10	30	20
USA	7	39 ± 12	25	60	35
All the countrie**s**	71	42 ± 23	0	90	90

Some suggested that it may be premature to conclude that lack of stroke awareness played a major role in the decrease in hospital admissions, but stroke education has to continue.^4^ We found that the major reason for the drop of stroke‐related admissions was that patients were not willing to come to the hospital due to a fear of virus infection, along with poor stroke awareness. While stroke awareness should not change dramatically in a short period of time, the issue of poor stroke awareness might have been magnified by the fear of acquiring COVID‐19. While improving stroke awareness should be a constant effort to remind people how to recognize stroke symptoms and seek help immediately, most of the efforts in stroke education for the public during the pandemic have decreased or totally stopped.^2^ We have been urging that stroke awareness and educational efforts, especially during the COVID‐19 pandemic, be enhanced.^5^ We must encourage people having stroke symptoms to call an emergency medical resource number immediately. Stroke self‐identification has become more critical due to social distancing and mask wearing. Facial drooping, a potential sign of stroke, will not be identified when one is wearing a mask. Slurred speech, another potential signs of stroke, will not be easily identified due to reduced verbal communication. Social distancing should not prevent checking on loved ones who may be at a high risk of stroke. A remote checking system with live high‐resolution video can be developed, based on individual situations. Stroke, especially ischemic stroke, is a silent killer since it generally does not cause severe pain or a feeling of impending doom. COVID‐19 can also be silent in the initial phases of infection. When these two silent killers coalesce, the outcome may be unfavorable. COVID‐19 patients could have neurological symptoms which could make stroke recognition more difficult.^6^ Our data indicate that while there was a drop in the number of thrombolysis and thrombectomy treatments, the thrombolysis and thrombectomy rates did not decrease, indicating that stroke patients will be well‐taken care of if they are sent to capable stroke centers during the COVID‐19 crisis.^2^ Implementation of multimodal computed tomography in a telestroke network has shown to be able to increase thrombolysis rate.^7^ This approach could potentially be useful during COVID‐19, in combination with chest computed tomography at the same time.^5^

We highly recommend initiating a robust international stroke awareness campaign, and experts across the world to unite to improve stroke awareness to reassure patients that they should seek care, since stroke is an emergent situation. The acronym of FAST (Face, Arm, Speech, and Time) for stroke awareness could be used in English‐speaking countries. The Stroke 120 strategy could be used in China,^8^ and a Stroke 112 strategy could be used in counties where 112 is used as emergency medical numbers.^9^ We have successfully demonstrated that using such novel tools changed the culture of stroke education and awareness across China.^10^ Different countries have had different peak periods during the COVID‐19 pandemic; thus, different strategies should be applied in order to be effective. The experience and evidence leaned and obtained from these countries, China, Japan, Korea, and some European countries where the epidemic peak period has passed should offer valuable guidance to other countries where the pandemic is still raging. In countries where COVID‐19 cases are still surging (eg,, USA, Brazil, India, Peru, Chile), it is critical to educate the public that the mortality rate for stroke is much higher than that for COVID‐19 and that they should call the emergency medical system for help immediately if a stroke is suspected. Stroke awareness educational efforts should be enhanced with electronic methods (phone calls with automatic messages like those ongoing by the American Heart Association/American Stroke Association, and online materials like this one developed at the University of Pennsylvania: https://www.pennmedicine.org/updates/blogs/neuroscience‐blog/2020/may/emergency‐care‐for‐strokes‐during‐covid19) during the pandemic, due to social distancing.

## CONFLICT OF INTEREST

The authors declare no conflict of interest.

## AUTHOR CONTRIBUTIONS

Dr Liu proposed the concept, analyzed data, and drafted and wrote the manuscript. Dr Zhao wrote the manuscript. Dr Fisher proposed the concept, collected data, analyzed data, and wrote the manuscript.
